# Comparison of ferulic acid content in Radix Angelicae Sinensis, Danggui-Buxue-Tang and Danggui-Sini-Tang

**DOI:** 10.3892/etm.2014.1547

**Published:** 2014-02-14

**Authors:** JIA-MEI YAO, LI-JIN YU, QIONG CHEN, ZE-QI CHEN, DONG-SHENG WANG, XIN-JIAN QIU, LIN-LIN ZHAO

**Affiliations:** 1 Department of Gerontology, Xiangya Hospital, Central South University, Changsha, Hunan 410008, P.R. China; 2 Laboratory of Ethnopharmacology, Institute of Integrated Traditional Chinese and Western Medicine, Xiangya Hospital, Central South University, Changsha, Hunan 410008, P.R. China

**Keywords:** ferulic acid, Radix Angelicae Sinensis, Danggui-Buxue-Tang, Danggui-Sini-Tang

## Abstract

The aim of the present study was to determine the ferulic acid (FA) content of Radix Angelicae Sinensis (AS), Danggui-Buxue-Tang (DBT) and Danggui-Sini-Tang (DST) using the same ultra performance liquid chromatography system and method. FA was eluted using an Acquity BEH C18 column (100×2.1 mm inner diameter; 1.7 μm). A mobile phase of methanol and 0.5% acetic acid was used and a flow rate of 0.3 ml/min was selected. The calibration curve exhibited a good linear regression (R^2^=0.9997). The inter- and intra-day precision measurements of FA ranged between 0.27 and 3.03% and the recovery ranged between 98.44 and 101.64% with relative standard deviation (RSD) values ≤4.73%. The method was reliable and simple. The results of the chromatographic analyses indicate that the FA contents of the DBT and DST decoctions were increased compared with that of AS due to the presence of other herbs.

## Introduction

Radix Angelicae Sinensis (AS), otherwise known as Danggui, has been used in traditional Chinese medicine for thousands of years and was first mentioned in Shenlong Bencao Jing (200–300 A.D., Han Dynasty) (1). AS is known for its clinical efficacy in the therapy of gynecological disease and has been named ‘female ginseng’ (1,2). Moreover, AS has been widely used for treating constipation, hepatic fibrosis, anemia and cardiovascular disease (3). In previous studies, AS has been shown to have numerous roles, including regulating the immune system and as an anticancer, anti-inflammatory and antioxidant agent (4–7). Over 70 compounds have been identified in AS and the components have been classified into two groups, including essential oils and water-soluble ingredients. Ferulic acid (FA) is one of the most abundant water-soluble ingredients and has been identified as the active component of AS (8).

Medicinal formulae containing AS are usually administered for benefiting vital energy, nourishing blood and regulating menstruation. Two examples are the formulae Danggui-Buxue-Tang (DBT) and Danggui-Sini-Tang (DST). One of the simplest formulae is DBT, which was first described in Neiwaishang Bianhuo Lun in 1247 A.D (9). As described, the DBT formula contained Radix Astragali and AS. DBT is usually administered to females for the treatment of menopausal symptoms. The formula regulates their health by invigorating vital energy and enriching the blood (10). DST, a traditional medicinal formula, was first recorded in Shanghan Lun (11) and is used to treat a variety of conditions, including poor extremity circulation, heart failure, shock and severe watery diarrhea. In particular, the formula is used for the therapy of infectious diseases that are complicated with hemodynamic instability (12,13).

FA was cited as the main index of components in AS, as described in the Pharmacopoeia of the People’s Republic of China 2010 (14). FA may be used to clear free radicals, reduce blood fat, regulate the immune system and decrease oxidative stress (15,16). FA is one of the most effective and active components of AS, DBT and DST.

In the present study, the FA content of AS, DBT and DST was determined by ultra performance liquid chromatography (UPLC) with the aim of investigating whether the FA content differed significantly between the formulae. Furthermore, the study investigated whether the FA content increased or decreased in decoctions that contained AS, compared with the FA level in AS alone.

## Materials and methods

### Reagents and materials

The DST formula contains seven traditional Chinese medicinal components: Radix Angelicae Sinensis (origin, Gansu; batch no. 2013010401), Radix Paeoniae Alba (origin, Zhejiang; batch no. 2013010402), Rumulus Cinnamomi (origin, Guangxi; batch no. 2013010403), Asari Radiix Etrhizoma (origin, Liaoning; batch no. 2013010404), Radix Glycyrrizae (origin, Neimeng; batch no. 2013010405), Medulla Tetrapanacis (origin, Shanxi; batch no. 2013010406 ) and Fructus Jujubae (origin, Xinjiang; batch no. 2013010407) in a weight ratio of 4:3:3:1:2:2:1. The DBT formula contains Radix Angelicae Sinensis (origin, Gansu; batch no. 2013010401) and Radix Astragali Mongolici (origin, Neimeng; batch no. 2013010408) in a weight ratio of 1:5. The herbs were purchased from the Xiangya Hospital Pharmacy (Changsha, China) and identified by director pharmacist Lei-Peng.

**Figure 1:**
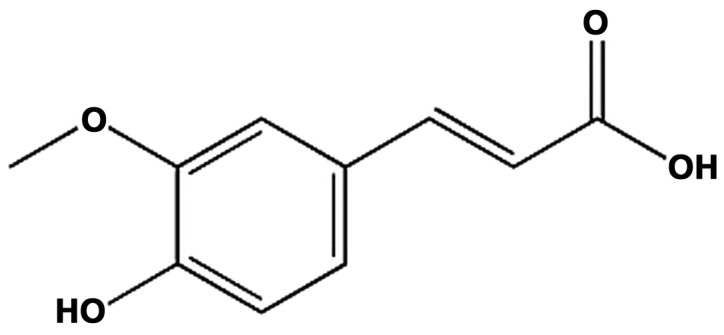
Structure of ferulic acid.

The FA standards (purity, >98%) were purchased from Chengdu Must Bio-Technology Co., Ltd. (Chengdu, China). The chemical structure of FA is shown in Fig 1. HPLC grade methanol and acetonitrile were purchased from Tedia Company, Inc. (Fairfield, OH, USA). Analytical grade acetic acid was obtained from Sinopharm Chemical Reagent Co. Ltd. (Shanghai, China). All other reagents were also analytical grade. Purified water was obtained using a Milli-Q purification system (Millipore Corporation, Billerica, MA, USA) and was used to prepare the mobile phases and sample solutions.

### Instruments

Chromatography was performed using a Waters Acquity UPLC system, with an automatic sample handling system (2695), diode array detector (2996), Empower 2.0 data processing software and Acquity BEH C18 column (100×2.1 mm internal diameter; 1.7 μm) (all from Waters Corporation, Milford, MA, USA).

### Chromatographic system

The mobile phases were solvent A (methanol) and solvent B (0.5% acetic acid). The gradient flow was as follows: 0 min, (A)/(B) = 0/100; 1 min, (A)/(B) = 18/82; 3 min, (A)/(B) = 28/72; and 4 min, (A)/(B) = 38/62. The column temperature was maintained at 40°C and the room temperature was 25°C. The volume of each injection was 6 μl.

### Preparation of standard solutions and calibration curves

The stock standard solution of FA was prepared by dissolving FA in methanol to obtain a 320 ng/μl solution. Next, seven concentrations of the standard solution were obtained by dilution of the stock solution. Working solutions and the stock solution were stored at 4°C. The standard curve was prepared using Empower software from the peak area of the seven concentrations determined by UPLC. The calibration curve ranged between 0.33 and 21.32 ng/μl. Further dilution of the lowest concentration working solution was performed to obtain a series of standard solutions to evaluate the limit of detection (LOD) and the limit of quantity (LOQ) of FA.

### Preparation of sample solutions

AS (20 g), DST (20 g AS, 15 g Radix Paeoniae Alba, 15 g Rumulus Cinnamomi, 5 g Asari Radiix Etrhizoma, 10 g Radix Glycyrrizae, 10 g Medulla Tetrapanacis and 5 g Fructus Jujubae) and DBT (20 g AS and 100 g Radix Astragali Mongolici) were immersed in distilled water (1:10; w/v) for 30 min and then boiled for 30 min. The boiling procedure was repeated twice. Each decoction was filtered and mixed together. Next, the solutions were lyophilized to acquire powdered DBT and DST. The powder was stored at 4°C. For UPLC analysis, the powders were dissolved in distilled water to provide solutions with final concentrations of 95 mg/ml AS, 300 mg/ml DBT and 164 mg/ml DST. A 100-μl sample of each solution was extracted with 900 μl methanol for 30 min by ultrasonic extraction. The extracted solution was centrifuged at 13,201 × g for 5 min. The supernatant was collected and filtered through a 0.22 μM filter and the final filtrate was analyzed by UPLC. A volume of 6 μl of each solution was injected into the column.

### Recovery test

Recovery tests are used to evaluate the accuracy of an analytical method. By spiking the standard solution and knowing the exact concentrations of the DBT and DST samples prior to abstraction, the recovery of FA was investigated. The following formula was used to calculate the percentage of recovery: Recovery (%) = (total content following spiking − original content in sample)/spiked content × 100.

## Results and Discussion

### Optimization of the chromatographic conditions

**Figure 2:**
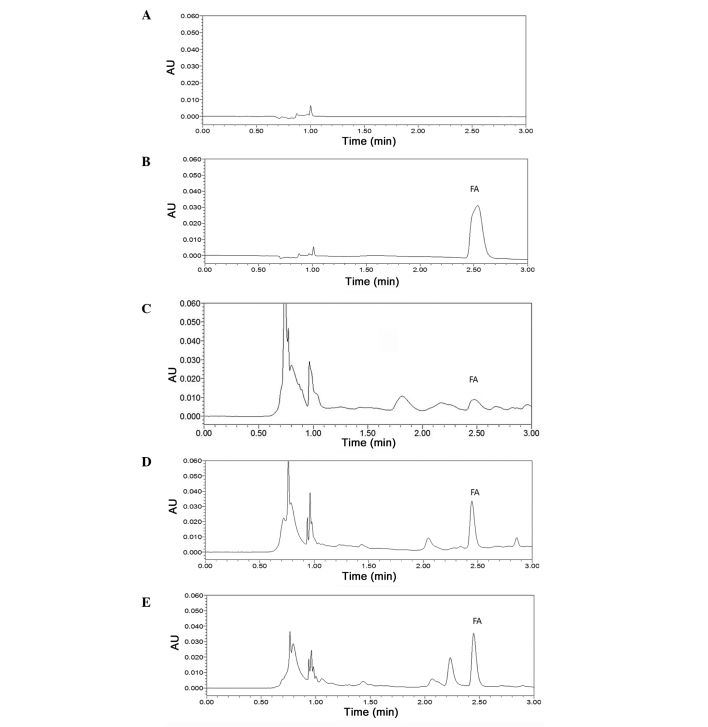
Typical chromatograms for determing the FA content at 320 nm in (A) the sample solvent (20% formic acid and methanol), (B) the standard solution, (C) AS, (D) DBT decoction and (E) DST decoction. AU, absorbance unit; FA, ferulic acid; AS, Radix Angelicae Sinensis; DBT, Dangui-Buxue-Tang; DST, Danggui-Sini-Tang.

In order to obtain an absolute peak and elute the target earlier, the selection of a chromatographic column, mobile phase and flow rate was important. In the study, various combinations of columns, mobile phases and flow rates were tested. By comparing the Acquity UPLC BEH shield RP18 column (2.1×100 mm; 1.7 μm) with the Acquity UPLC BEH C18 column (2.1×100 mm; 1.7 μm), the latter was selected for this study. Mobile phases consisting of methanol with water, methanol with 0.5% acetic acid, acetonitrile with water and acetonitrile with 0.5% acetic acid were applied under various gradient elution modes and flow rates. Following a number of trials, the Acquity UPLC BEH C18 column, a mobile phase of methanol with 0.5% acetic acid, a flow rate of 0.3 ml/min and gradient elution modes were selected as the suitable chromatogram conditions. Under these conditions, the desired separation was achieved within 5 min, the peak shape was improved and peak tailing was inhibited. The wavelength of the PAD detector was selected according to the maximum UV absorption wavelength of FA which was 320 nm. Typical chromatograms of the blank solvent (20% formic acid and methanol), standard solution, AS, DBT and DST are shown in Fig 2.

### Optimization of the extraction conditions

Protein precipitation was the main method used to extract the objective from the decoction and the selection of the solvent used for precipitation was important. In the study, the solvents tested were methanol, 5% formic acid with methanol, 10% formic acid with methanol, 20% formic acid with methanol, 5% NaHCO_3_ with methanol, 10% NaHCO_3_ with methanol and 20% NaHCO_3_ with methanol. It was identified that 10% formic acid and methanol were the most effective solvents for the extraction of FA from AS, DBT and DST. The FA content of RAS, DBT and DST was determined using the same method, and five solutions of every sample were determined at the same time. The results are shown in Table I.

### Calibration curve

A calibration curve was prepared with the data from seven concentrations of the standard solution of FA. The calibration curve had the following equation: y = 2E − 05x + 0.1263. The calibration data exhibited a good linear regression (R^2^=0.9997), ranging between 0.33 and 21.32 ng/μl. The LOD [signal to noise ratio (S/N), 3) and LOQ (S/N, 10) values were calculated to be 0.04 and 0.128 ng/μl, respectively.

### Precision and accuracy

Method precision was assessed by intra- and inter-day assays. Intra- and inter-day precisions were determined by testing three concentrations of the standard solution five times in one day and once a day on three consecutive days. Relative standard deviation (RSD) values were considered to indicate the measurement precision. As shown in Table II, the RSD values for the evaluation of intra- and inter-day precision ranged between 0.27 and 3.03%.

### Stability

Stability analysis was performed by evaluating the sample solutions at 4°C at various time points (0, 4, 10, 16 and 24 h). The RSD values of FA concentration were ≤1.63%, as shown in Table III. The results indicate that FA was stable for 24 h at 4°C.

### Repeatability and recovery

Extraction of samples and analyses were repeated using the same method and chromatography system. The peak area of FA was obtained and the RSD values of repeatability ranged between 1.15 and 3.74%, as shown in Table IV. As the data indicates, the present method for testing the content of FA was reproducible. The accuracy tests were accomplished by a recovery test. In the study, the average recoveries of FA ranged between 98.44 and 101.64%, with RSD values ≤4.73% (Table V). The results indicate that the method was reliable and accurate for measuring the content of FA.

### Conclusion

In conclusion, using a simple and reliable UPLC method, the FA content of AS, DBT and DST was accurately determined. As is commonly understood, differences in active compounds exist between traditional medicines used singly and in formulations, resulting in varying clinical effects. From the present study, a comparison of the FA content of AS with that of the combination of herbs in the DBT and DST formulae shows an increased FA content in the combinations.

## Figures and Tables

**Table I:** FA content in 95 mg/ml AS, 300 mg/ml DBT and 164 mg/ml DST.

	FA content, ng/μl						
		
Source	Group 1	Group 2	Group 3	Group 4	Group 5	Mean	RSD, %
AS	1.23	1.28	1.25	1.36	1.29	1.28	4.00
DBT	2.06	2.04	2.06	2.07	1.95	2.04	2.42
DST	2.24	2.20	2.09	2.09	2.22	2.17	3.36

Five solutions of each sample were tested. FA, ferulic acid; AS, Radix Angelicae Sinensis; DBT, Danggui-Buxue-Tang; DST, Danggui-Sini-Tang.

**Table II:** Measurement precision of the UPLC method for the detection of FA.

	Precision			
	
	Intra-day, n=5		Inter-day, n=5	
		
Nominal concentration, ng/μl	Mean ± SD, ng/μl	RSD, %	Mean ± SD, ng/μl	RSD, %
10.66	10.66±0.03	0.27	10.35±0.28	2.67
2.67	2.75±0.02	0.89	2.52±0.08	3.03
0.67	0.71±0.01	1.18	0.62±0.02	2.43

UPLC, ultra performance liquid chromatography; FA, ferulic acid; RSD, relative standard deviation.

**Table III:** Stability of FA at 4°C (n=5).

	FA content, ng/μl					
		
Source	0 h	4 h	10 h	16 h	24 h	RSD, %
AS	1.28	1.27	1.27	1.24	1.30	1.63
DBT	2.04	2.05	2.08	2.05	2.10	1.23
DST	2.17	2.15	2.16	2.12	2.13	0.94

FA, ferulic acid; AS, Radix Angelicae Sinensis; DBT, Danggui-Buxue-Tang; DST, Danggui-Sini-Tang.

**Table IV:** Repeatability of the method.

	FA content, ng/μl						
		
Source	Test 1	Test 2	Test 3	Test 4	Test 5	Mean	RSD, %
AS	1.25	1.29	1.24	1.30	1.28	1.28	2.04
DBT	2.08	2.09	2.08	2.03	2.07	2.07	1.15
DST	2.07	2.09	2.12	2.27	2.17	2.14	3.74

FA, ferulic acid; AS, Radix Angelicae Sinensis; DBT, Danggui-Buxue-Tang; DST, Danggui-Sini-Tang.

**Table V:** Recovery of FA.

Source	Original content (ng/μl)	Spiked content (ng/μl)	Total content (ng/μl)	Recovery, %	RSD, %
AS	32.0	21.32	53.67	101.64	4.73
DBT	81.6	85.28	165.55	98.44	2.92
DT	86.8	85.28	171.37	99.17	2.18

FA, ferulic acid; AS, Radix Angelicae Sinensis; DBT, Danggui-Buxue-Tang; DST, Danggui-Sini-Tang.
